# Serum creatinine/cystatin C ratio is a systemic marker of sarcopenia in patients with gastrointestinal stromal tumours

**DOI:** 10.3389/fnut.2022.963265

**Published:** 2022-09-02

**Authors:** Ping’an Ding, Honghai Guo, Chenyu Sun, Shuya Chen, Peigang Yang, Yuan Tian, Scott Lowe, Qun Zhao

**Affiliations:** ^1^The Third Department of Surgery, The Fourth Hospital of Hebei Medical University, Shijiazhuang, China; ^2^Hebei Key Laboratory of Precision Diagnosis and Comprehensive Treatment of Gastric Cancer, Shijiazhuang, China; ^3^AMITA Health Saint Joseph Hospital Chicago, Chicago, IL, United States; ^4^Newham University Hospital, London, United Kingdom; ^5^College of Osteopathic Medicine, Kansas City University, Kansas City, MO, United States

**Keywords:** serum creatinine, serum cystatin C, serum Cr/CysC ratio, sarcopenia, gastrointestinal stromal tumors

## Abstract

**Background:**

It is well known that sarcopenia is a common risk factor in patients with gastrointestinal tumours, which may negatively affect the clinical outcome and prognosis. Recent studies suggest that serum creatinine-cystatin C (Cr/CysC) ratio may be associated with sarcopenia, but this association lacks sufficient evidence in patients with gastrointestinal stromal tumours (GIST). Therefore, this study aimed to investigate whether the Cr/CysC ratio was associated with sarcopenia and recurrence-free survival (RFS) in patients with GIST.

**Materials and methods:**

The study retrospectively analysed 413 patients with GIST who underwent surgical resection from January 2016 to January 2020. The serum Cr/CysC ratio was determined as a proxy for sarcopenia by comparing it with various biomarkers and Cox multifactorial analysis was used to determine the relationship between Cr/CysC ratio and prognosis.

**Results:**

Serum Cr/CysC was positively correlated with skeletal muscle area (SMA) (*r* = 0.256, *p* < 0.001), skeletal muscle index (SMI) (*r* = 0.300, *p* < 0.001), and hand grip strength (HGS) (*r* = 0.251, *p* < 0.001). The area under the receiver operator characteristic curve for sarcopenic subjects with serum Cr/CysC ratio was significantly greater than other biomarkers (Cr/CysC: 0.840, CysC: 0.732, Cr: 0.518). The optimal cut-off value for Cr/CysC was 0.65, and patients in the high Cr/CysC group had a higher 3-year recurrence-free survival (RFS) than those in the low Cr/CysC group (92.72 vs. 72.46%, *p* < 0.001). Cox multifactorial analysis found that the Cr/CysC ratio was an independent risk factor for RFS in GIST patients (HR = 2.143, 95% CI: 1.431–5.459, *p* = 0.011).

**Conclusion:**

Serum Cr/CysC ratio has satisfactory and comparable diagnostic accuracy, and prognostic value for sarcopenia in patients with GIST. Therefore, it can be a simple and practical clinical tool to screen sarcopenia in GIST patients. However, further studies are required to validate these findings.

## Introduction

Gastrointestinal stromal tumours (GIST) are the most common type of mesenchymal tumour, occurring mainly in the stomach (40–60%) followed by the small intestine (20–30%) (1, 2). Recent studies have found that sarcopenia is common in GIST patients, with an incidence rate of 26.2%, especially for locally advanced GIST patients with larger tumour diameter and special tumour locations (3, 4). Sarcopenia is an age-related syndrome associated with decreased muscle mass, strength, and function (5). In recent years, sarcopenia has received extensive attention as not only a predictor of oral imatinib tolerance in GIST patients but also an unfavourable factor affecting prognosis (3, 4). Therefore, detection of sarcopenia at an early stage in GIST patients specifically in locally advanced GIST patients has more benefits.

Currently, the published international consensus diagnosis of sarcopenia considers that it is necessary to measure muscle function and to evaluate the patient’s physical performance, in addition to the assessment of muscle mass (6–8). Several diagnostic methods have been proposed for the measurement of muscle mass in previous studies, including dual-energy X-ray imaging (DXA) measurement of the appendicular skeletal muscle mass index (ASMI), computed tomography (CT) and magnetic resonance imaging (MRI) to measure the cross-sectional area of the third lumbar vertebrae muscle, and bioelectrical impedance analysis (BIA) to measure the whole-body skeletal muscle mass (SMM) or the appendicular skeletal muscle mass (ASM) (9, 10). Muscle function is usually tested by hand grip strength (HGS) or chair stand/rise test (5 sit-ups), and physical performance can be measured by pace or short physical performance battery (SPPB), timed up-and-go test (TUG) and 6-m usual gait speed (10–12). Nonetheless, these traditional methods of assessing sarcopenia are complex and not feasible in clinical practice and it is urgent to find a simple and reliable serum diagnostic marker for the assessment of sarcopenia in GIST patients.

It is well known that serum creatinine (Cr) and cystatin C (CysC) are commonly used to assess renal function in cancer patients. In recent years, researchers have found that the ratio of Cr and Cyc in peripheral blood can be used to predict muscle mass. Cr is an index reflecting the function of glomerular filtration, which is mainly affected by the metabolism of muscle tissue in the body and is mainly excreted by glomerular filtration (13, 14). Meanwhile, CysC is uniquely derived from all nucleated cells and is not influenced by muscle mass volume (15, 16). Consequently, in patients with skeletal muscle mass loss, serum creatinine levels appear to decrease, whereas cystatin C levels do not decrease significantly. Based on these observations, the serum creatinine/cystatin C (Cr/CysC) ratio can further predict muscle mass and has been demonstrated in patients with oesophageal cancer (17), gastric cancer (18), non-small cell lung cancer (19), and chronic obstructive pulmonary disease (20). However, the association of Cr/CysC ratio with sarcopenia in GIST patients has not been reported. In the meantime, the evidence supporting the predictive value of the Cr/CysC ratio for sarcopenia has not been validated by studies with a large sample size.

Therefore, the aim of this study was to investigate whether Cr/CysC can be utilised for screening sarcopenia in GIST patients and to determine its optimal cut-off value and impact on recurrence-free survival (RFS) in GIST patients.

## Materials and methods

### Study design and participants

This retrospective cohort study was conducted in the Fourth Hospital of Hebei Medical University. The clinical data of GIST patients who were consecutively admitted to the Three Departments of Surgery were analysed from January 2016 to January 2020. The inclusion criteria were as follows: age ≥ 18 years, pathological diagnosis of GIST, treatment with surgery, serum Cr and CysC measured within 1 week before surgery and computed tomography (CT) of the abdomen and pelvis within 2 weeks. Exclusion criteria were as follows: receiving targeted therapy, concurrent presence of other types of tumour, diagnosis of metastatic GIST, incomplete clinical data, reduced preoperative glomerular filtration, and absent abdominal and pelvic CT images. A total of 449 patients were enrolled in the study, of whom 36 were excluded for the following reasons: 18 patients had missing abdominal and pelvic CT images, 10 patients received preoperative imatinib-targeted therapy, and 8 patients had impaired preoperative glomerular filtration function. Ultimately, data from 413 patients were analysed. Informed consents were obtained from all participants and the study protocol was ethically approved by the biomedical research ethics committee of the Fourth Hospital of Hebei Medical University (Ethics number: 2019049).

### Laboratory measurements

In all patients, within 1 week before the procedure, peripheral venous blood was collected after fasting for 8 h. The blood creatinine level and CysC level were measured using an automated haematology analyser (Beckman Coulter AU5800). According to previous research reports (21–24), the Cr/CysC ratio was calculated as follows: the Cr/CysC ratio = [(serum Cr{mg/dL}/serumCysC{mg/L}) × 100].

### Measurement of muscle mass and strength

Abdominal and pelvic CT images of all recruited patients before the surgery were analysed retrospectively. Numerous studies have confirmed that the area of skeletal muscle and adipose tissue in CT cross-sectional images of the L3 vertebral level scan performed in the supine position is closely related to the total body muscle and fat mass (25, 26). Therefore, skeletal muscle area at the level of lumber vertebral L3 was measured in GIST patients.

The 5-mm flat-scan images were uploaded to the Picture Archiving and Communication System (PACS, SIEMENS SOMATOM) after image acquisition. The following tissue Hounsfield unit (HU) thresholds were used: skeletal muscle had an attenuation range of -29 to 150 HU (27). The software evaluated and measured the pixel area of the corresponding area of skeletal muscle attenuation to obtain the skeletal muscle area (SMA). The skeletal muscle mass index (SMI) was then calculated based on the equation: SMI (cm^2^/m^2^) = SMA/height^2^.

The handgrip strength (HGS) was evaluated using a Camry dynamometer (EH101; Xiangshan Company, Guangdong, China), both hands were alternately measured twice, and the maximum value was used for further analysis.

### Diagnostic criteria for sarcopenia

The definition of sarcopenia in this study was assessed using the European working group on sarcopenia in older people (EWGSOP) (6) and the Asian working group on sarcopenia (AWGS) (7) consensus. Specific criteria for the definition of sarcopenia include: SMI < 40.8 cm^2^/m^2^ in men and SMI < 34.9 cm^2^/m^2^ in women as muscle loss, and HGS < 26 kg in men and HGS < 18 kg in women as sarcopenia.

### Follow-up of participants

Recurrence-free survival (RFS) was defined as the time from the start of initial recruitment to the date of documented relapse or death from any cause during the follow-up, and the follow-up deadline for this study was May 31, 2022. All patients were recommended to have an enhanced CT scan of the abdomen and pelvis every 3 months for the first 3 years postoperatively and every 6 months for the 4th to 5th postoperative years. Follow-up methods mainly included telephone encounters, outpatient visits, and hospitalisation.

### Statistical analysis

All statistical analyses were performed using IBM SPSS Statistics for Windows, version 26.0 (IBM Corp, Armonk, NY, United States) and MedCalc Statistical Software v15.2 (MedCalc Software bvba, Ostend, Belgium). Categorical variables were expressed as numbers and proportions, using the *X*^2^ test or Fisher’s exact test. Continuous variables were expressed as mean with standard deviation (SD) or median with interquartile range (IQR). All continuous data in this study were non-normally distributed according to the Kolmogorov–Smirnov test, so the Wilcoxon rank-sum test was used to compare differences between groups. Scatter plots and Pearson’s correlation coefficient were used to assess linear correlations. Multivariate logistic regression was used to analyse the effect of various factors on the prevalence of sarcopenia. The subject receiver operating characteristic (ROC) curve and area under the curve (AUC) were used to assess the ability of the Cr/CysC ratio, CysC and Cr to screen for sarcopenia. Differences in subject working characteristic (ROC) curves were compared using DeLong test. The Youden index (sensitivity + specificity-1) was calculated to determine the optimal cut-off point for determining the Cr/CysC ratio in men and women. Kaplan–Meier curves were performed to estimate RFS, and a log-rank test was used to compare the difference between groups. Cox regression models were used to identify independent prognostic factors. A *P*-value < 0.05 was considered to show statistical significance.

## Results

### Patients characteristics

Table 1 summarises the demographic information and pathological characteristics of the 413 patients with GIST. The majority of patients had a tumour site in the stomach and the mean age of the participants was 59.7 ± 10.3 years. According to the 2008 edition of the National Institutes of Health (NIH) stromal tumour risk classification criteria (28), more than half of the patients in this study were intermediate-risk (37.29%) and high-risk (22.28%). In addition, these patients were detected for c-kit and PDGFRA gene after operation. Based on the recommendations of the Chinese consensus guidelines for the diagnosis and treatment of gastrointestinal stromal tumours, except for patients with PDGFRA exon 18 D842V mutation, exon 9 mutation, and wild-type GIST, the remaining patients received imatinib 400 mg/day adjuvant therapy. The mean serum creatinine at admission was 0.65 ± 0.25 and the mean serum CysC was 0.89 ± 0.31.

**TABLE 1:** Characteristics of patients included in the study.

Variables	All patients (*N* = 413)	Sarcopenia (*N* = 87)	Non-sarcopenia (*N* = 326)	*p*
Age (years)	59.7 ± 10.3	59.9 ± 10.6	59.3 ± 9.2	0.376
Weight (kg)	63.3 ± 11.6	60.6 ± 11.8	62.1 ± 10.7	0.017
BMI (kg/m^2^)	25.0 ± 2.9	23.9 ± 3.0	25.2 ± 2.6	0.002
Sex, *n* (%)				0.689
Male	201 (48.67)	44 (50.57)	157 (48.16)	
Female	212 (51.33)	43 (49.43)	169 (51.84)	
Tumour location, *n* (%)				0.114
Stomach	253 (61.26)	44 (50.57)	209 (64.11)	
Duodenum	25 (6.05)	6 (6.90)	19 (5.83)	
Intestine	76 (18.40)	19 (21.84)	57 (17.48)	
Colon	29 (7.02)	7 (8.05)	22 (6.75)	
Mesentery	30 (7.26)	11 (12.64)	19 (5.83)	
Tumour size (cm)	5.3 ± 4.8	6.7 ± 3.2	5.0 ± 4.4	0.030
Nuclear mitotic figure (50HPF), *n* (%)				< 0.001
< 5	149 (36.08)	11 (12.64)	138 (42.33)	
6–10	236 (57.14)	65 (74.71)	171 (52.45)	
> 10	28 (6.78)	11 (12.64)	17 (5.21)	
Risk classification				< 0.001
Very low risk	23 (5.57)	0 (0)	23 (100.00)	
Low risk	144 (34.87)	10 (11.49)	134 (41.10)	
Middle risk	154 (37.29)	32 (36.78)	122 (37.42)	
High risk	92 (22.28)	45 (51.72)	47 (14.42)	
c-kit exons, *n* (%)				0.382
Positive	268 (64.89)	53 (60.92)	215 (65.95)	
Negative	145 (35.11)	34 (39.08)	111 (34.05)	
PDGFRA exons, *n* (%)				0.142
Positive	112 (27.12)	29 (33.33)	83 (25.46)	
Negative	301 (72.88)	58 (66.67)	243 (74.54)	
Haemoglobin (g/L)	116.1 ± 11.1	114.9 ± 9.7	116.5 ± 11.4	0.207
Handgrip strength (Kg)	24.4 ± 4.8	19.6 ± 3.1	25.6 ± 4.4	< 0.001
Albumin (g/L)	42.3 ± 3.4	38.7 ± 3.3	42.4 ± 3.4	0.008
Prealbumin	236.4 ± 28.2	220.2 ± 25.2	236.2 ± 28.1	0.005
Total protein	62.9 ± 6.7	62.9 ± 6.3	63.0 ± 6.9	0.736
SMA (cm^2^)	106.6 ± 16.3	92.5 ± 11.5	110.4 ± 15.2	< 0.001
SMI (cm^2^/m^2^)	38.2 ± 4.4	33.5 ± 2.9	39.5 ± 3.9	< 0.001
Serum Cr (mg/dL)	0.65 ± 0.25	0.57 ± 0.31	0.70 ± 0.23	< 0.001
Serum CysC (mg/L)	0.89 ± 0.31	0.87 ± 0.24	0.90 ± 0.33	0.606
Cr/CysC ratio	0.77 ± 0.33	0.47 ± 0.23	0.83 ± 0.31	< 0.001

BMI, body mass index; Cr, creatinine; CysC, cystatin C; Cr/CysC ratio, serum creatinine/cystatin C ratio; SMA, skeletal muscle mass; SMI, skeletal muscle index. Values are presented as mean (SD) unless otherwise noted.

### Correlation between clinical factors for sarcopenia

In this study, a total of 87 patients (21.07%) had sarcopenia, including 44 (50.57%) men and 43 (49.43%) women. The characteristics of patients with sarcopenia compared to those without sarcopenia are shown in Table 1. To determine whether the Cr/Cys C ratio was associated with sarcopenia, a linear regression analysis was performed to identify the predictors of sarcopenia. In univariate analyses, it was found that patients in the sarcopenic group had lower weight, BMI, albumin, prealbumin, Cr/CysC ratio, and HGS, compared to those without sarcopenia. As shown in Table 2, multivariate analysis showed that Cr/CysC ratio (OR = 4.722, 95% CI: 2.312–33.871, *p* = 0.002) remained as an independent predictor of sarcopenia.

**TABLE 2:** Multivariate analyses of the clinicopathological characteristics for sarcopenia.

Variables	OR	95% CI	*P*-value
BMI (< 25.0 Kg/m^2^/≥ 25.0 Kg/m^2^)	3.452	1.452–13.256	0.013
Tumour size (≥ 5.3 cm/< 5.3 cm)	2.891	1.302–8.192	0.030
Risk classification (High/No-high)	4.568	2.312–21.456	0.009
Serum Cr (< 0.65 mg/dL/≥ 0.65 mg/dL)	1.092	0.782–7.980	0.302
Cr/CysC ratio (< 0.77/≥ 0.77)	4.722	2.312–33.871	0.002

BMI, body mass index; Cr, creatinine; CysC, cystatin C; Cr/CysC ratio, serum creatinine/cystatin C ratio; OR, odds ratio; CI, confidence interval.

### Associations between Cr/CysC ratio and sarcopenia

As shown in Table 3, serum CysC was not significantly correlated with SMI (*r* = -0.0405, *p* = 0.359), SMA (*r* = -0.091, *p* = 0.064), or HGS (*r* = 0.071, *p* = 0.152), whereas serum Cr was positively correlated with SMI (*r* = 0.206, *p* < 0.001), SMA (*r* = 0.161, *p* = 0.001), and HGS (*r* = 0.234, *p* < 0.001). In addition, the Cr/CysC ratio correlated more strongly with SMI (*r* = 0.300, *p* < 0.001), SMA (*r* = 0.256, *p* < 0.001), and HGS (*r* = 0.251, *p* < 0.001) than between Cr and SMA, SMI, and HGS, respectively.

**TABLE 3:** Correlation coefficients between biomarkers and clinical factors.

Clinical factors	Serum Cr/CysC		Serum Cr		Serum CysC	
			
	*r*	*P*-value	*r*	*P*-value	*r*	*P*-value
Handgrip strength	0.251	<0.001	0.234	<0.001	0.071	0.152
BMI	-0.018	0.708	0.048	0.330	0.095	0.053
Haemoglobin	0.057	0.251	0.020	0.688	0.007	0.889
Prealbumin	0.011	0.826	–0.019	0.696	–0.027	0.588
Albumin	0.106	0.032	0.094	0.056	–0.006	0.897
Total protein	0.027	0.587	0.037	0.455	0.008	0.868
SMA	0.256	<0.001	0.161	0.001	–0.091	0.064
SMI	0.300	<0.001	0.206	<0.001	–0.045	0.359

BMI, body mass index; SMA, skeletal muscle area; SMI, skeletal muscle mass index.

### Diagnostic value of Cr/CysC ratio for detecting sarcopenia

**FIGURE 1:**
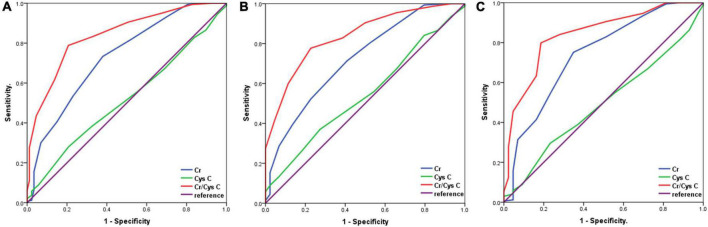
Receiver operator characteristic curves for the analysis of serum Cr/CysC ratio, CysC and Cr levels for the diagnosis of sarcopenia according to gender. **(A)**, All patients; **(B)**, Male patients; **(C)**, Female patients.

We analysed the diagnostic value of serum Cr, CysC, and Cr/CysC ratio for sarcopenia by using ROC curves, and the validity of each biomarker as a predictor of sarcopenia was examined by using DeLong test (Figure 1). The patients were divided into two groups according to gender. Serum Cr/CysC ratio, CysC, and Cr AUC were 0.838 (95% CI: 0.776–0.899), 0.543 (95% CI: 0.452–0.635), and 0.723 (95% CI: 0.638–0.808) in men respectively and were 0.841 (95% CI: 0.775–0.907), 0.491 (95% CI: 0.400–0.583), and 0.739 (95% CI: 0.652–0.827) in women respectively. Comparative analysis by DeLong test showed that the AUC of Cr/CysC ratio in both groups was significantly greater than that of CysC and Cr (*p* = 0.021 and *p* = 0.016). Moreover, a serum Cr/CysC cut-off value of 0.65 for all patients was calculated using the Youden-index, and the same cut-off values were obtained for the subgroup analysis of male and female patients. Based on the cut-off values, the sensitivity, specificity, positive predictive value, and negative predictive value were calculated as 0.777, 0.773, 49.28, and 92.42%, respectively in the male group. In the group, the sensitivity, specificity, positive predictive value and negative predictive value were 0.799, 0.814, 48.22, and 94.41%, respectively (Table 4). Sarcopenia was detected in 6.55% (18/275) of patients with a high serum Cr/CysC ratio (≥ 0.65) and 50.00% (69/138) of patients with a low serum Cr/CysC ratio (< 0.65) (*p* < 0.001).

**TABLE 4:** Serum Cr/CysC ratio, CysC and Cr levels for the diagnosis of sarcopenia.

Variables	AUC (95% CI)	Optimal cut-off	Sensitivity	Specificity	PPV (%)	NPV (%)
Cr/CysC ratio						
Overall	0.840 (0.795–0.885)	0.65	0.788	0.793	50.00	93.45
Men	0.838 (0.776–0.899)	0.65	0.777	0.773	49.28	92.42
Female	0.841 (0.775–0.907)	0.65	0.799	0.814	48.22	94.41
Cys C						
Overall	0.518 (0.453–0.583)	1.05	0.279	0.793	20.00	78.71
Men	0.543 (0.452–0.635)	0.95	0.369	0.727	26.47	79.04
Female	0.491 (0.400–0.583)	1.05	0.296	0.767	13.89	78.41
Cr						
Overall	0.732 (0.671–0.792)	0.75	0.405	0.851	30.59	89.69
Men	0.723 (0.638–0.808)	0.75	0.395	0.864	31.19	89.13
Female	0.739 (0.652–0.827)	0.75	0.414	0.837	30.00	90.20

AUC, area under the curve; Cr/CysC, serum creatinine/cystatin C; NPV, negative predictive value; PPV, positive predictive value.

### Association between Cr/CysC ratio and recurrence-free survival

**FIGURE 2:**
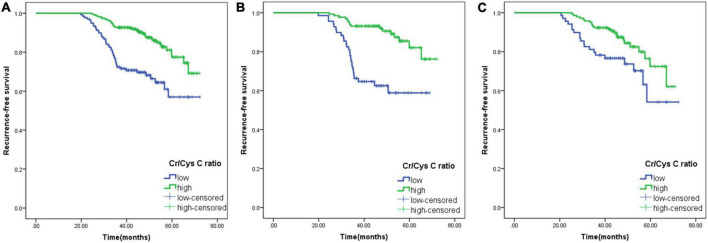
Comparison of the prognosis of patients with low and high serum creatine (Cr)/cystatin C (CysC) ratios. **(A)**, All patients; **(B)**, Male patients; **(C)**, Female patients. Cr/CysC < 0.65 was defined as low Cr/CysC; Cr/CysC > 0.65 was defined as high Cr/CysC.

The median follow-up time for all patients was 7 months (range: 0–23 months), and the 3-year RFS was 85.96%. The Kaplan–Meier curve was plotted using the calculated optimal cut-off value of serum Cr/CysC ratio, and it was found that the 3-year RFS of patients in the low serum Cr/CysC ratio group was significantly lower than that in the high Cr/CysC ratio group (72.46 vs. 92.72%, *p* < 0.001). The same results were obtained when subgroup analysis was carried out according to gender (male, 66.67 vs. 93.18%, *p* < 0.001; female, 78.26 vs. 92.31%, *p* < 0.001) (Figure 2). We performed Cox regression using age, BMI, pTNM stage and tumour size for multivariate survival analysis, and the results showed that the Cr/CysC ratio was an independent prognostic factor affecting RFS in GIST patients (HR = 2.143, 95% CI: 1.431–5.459, *p* = 0.011) (Table 5).

**TABLE 5:** Univariate and multivariate analyses of the clinicopathological characteristics for RFS.

Independent factor	Univariate analysis			Multivariate analysis		
		
	Hazard ratio	95% CI	*P*-value	Hazard ratio	95% CI	*P*-value
Sex						
Female	1.000	reference				
Male	1.014	0.663–1.551	0.950			
Age (years)						
< 60	1.000	reference				
≥ 60	1.178	0.769–1.805	0.573			
BMI (kg/m^2^)						
< 25.0	1.000	reference				
≥ 25.0	1.366	0.893–2.089	0.152			
Tumour location						
Stomach	1.000	reference				
No-stomach	1.135	0.731–1.763	0.265			
Nuclear mitotic figure (50HPF)						
< 5	1.000	reference		1.000	reference	
6–10	3.858	1.126–13.210	< 0.001	2.537	1.435–7.855	0.013
> 10	5.002	2.514–9.952	< 0.001	4.431	2.670–8.896	0.002
Tumour size (cm)						
< 10.0	1.000	reference		1.000	reference	
≥ 10.0	3.218	1.781–7.723	0.002	2.679	1.522–6.692	0.018
Risk classification			0.001			0.001
Very low-Low risk	1.000	reference		1.000	reference	
Middle risk	2.185	1.200–3.981	0.001	2.339	1.332–5.705	0.003
High risk	7.259	4.067–12.950	< 0.001	5.671	2.672–10.296	0.002
c-kit exons						
Positive	1.000	reference				
Negative	1.350	0.870–2.095	0.056			
PDGFRA exons						
Positive	1.000	reference				
Negative	1.307	0.806–2.118	0.082			
Sarcopenia						
No	1.000	reference		1.000	reference	
Yes	5.716	3.161–10.340	< 0.001	3.325	1.651–7.848	0.004
Cr/CysC ratio						
≥ 0.65	1.000	reference		1.000	reference	
< 0.65	2.854	1.788–4.554	< 0.001	2.143	1.431–5.459	0.011

BMI, body mass index; Cr, creatinine; CysC, cystatin C; Cr/CysC ratio, serum creatinine/cystatin C ratio; CI, confidence interval.

## Discussion

This study shows that the serum Cr/CysC ratio in GIST patients is positively correlated with muscle mass and grip strength. In addition, the serum Cr/CysC ratio was also an independent predictor of the development of sarcopenia after adjusting for potential confounders, it indicates a high predictive value for sarcopenia in GIST patients. An optimal serum Cr/CysC cut-off value of 0.65 (specificity: 0.793; sensitivity: 0.788) was calculated, the percentage of sarcopenia in the low serum Cr/CysC ratio group (50.00%) was significantly higher than that in the high serum Cr/CysC ratio group (6.55%) based on the cut-off value. Meanwhile, Cox multivariate regression analysis revealed that serum Cr/CysC ratio was an independent predictor of RFS in GIST patients. Therefore, these findings suggest that serum Cr/CysC ratio can be used as an alternative biomarker for screening sarcopenia in GIST patients.

According to the published consensus reports of EWGSOP (6) and AWGS (7), it is recommended to measure SMI at L3 level, grip strength, and gait speed for the diagnosis of sarcopenia. However, it is difficult to assess these measurements in all GIST patients in daily practice. Numerous studies have found changes in serum Cr are mainly affected by skeletal muscle metabolism, which is mainly manifested by lower creatinine levels in patients with reduced muscle mass (29). At present, although circulating creatinine is affected by chronic diseases, age, and pathological conditions such as protein-losing diseases, studies have confirmed that serum Cr at steady state has been used as a surrogate indicator for muscle mass measurement (30, 31). Therefore, in some clinical situations, low serum Cr levels can be considered as a proxy for muscle wasting. In contrast, serum CysC is a small, non-ionic protein that is not affected by muscle metabolism and is produced at a constant rate (32). Based on these characteristics, the calculation of the Cr/CysC ratio can be used to assess skeletal muscle metabolism and further screen patients for sarcopenia. Since its introduction, the serum Cr/CysC ratio has been widely evaluated as a valid, low-cost, and reproducible biomarker in a variety of diseases, such as gastric cancer (18), oesophagal cancer (17), idiopathic pulmonary fibrosis (33).

In this study, it demonstrated a positive correlation between serum Cr/CysC ratios and the consensus recommended measures of SMI (*r* = 0.300, *p* < 0.001), SMA (*r* = 0.256, *p* < 0.001), and HGS (*r* = 0.251, *p* < 0.001), which is consistent with previous studies (32, 33). In addition, this correlation was stronger than the correlation between the single indicator Cr and SMI (*r* = 0.206, *p* < 0.001), SMA (*r* = 0.161, *p* = 0.001), and HGS (*r* = 0.234, *p* < 0.001). The study showed that Cr/CysC ratio (OR = 4.722, 95% CI: 2.312–33.871, *p* = 0.002) remained an independent predictor of sarcopenia in GIST patients after adjustment of BMI, tumour size, risk classification and Cr by multivariate logistic regression analysis, which is consistent with previous findings in gastric cancer patients (18). In addition, a retrospective study by Zheng et al. found that the ratio of Cr/CysC had a good diagnostic effect on sarcopenia in both men (AUC = 0.732) and women (AUC = 0.754) with oesophageal cancer (17). Similar results were obtained in the present study, the serum Cr/CysC ratio was a useful predictor of sarcopenia compared with other biomarkers (e.g., serum Cr, CysC), and subgroup analysis performed according to gender found that the serum Cr/CysC ratio could also predict sarcopenia in GIST patients. In addition, to the best of our knowledge, no previous studies explored the optimal cut-off value of the Cr/CysC ratio for sarcopenia in GIST patients, while our study found a Youden index of 0.65 for all GIST patients, as well as male and female patients.

Currently, the relationship between the Cr/CysC ratio measured at the first diagnosis of cancer patients and the prognosis of cancer patients has attracted increasing attention. A retrospective study of 3,060 cancer patients found that an increase in the Cr/CysC ratio at diagnosis was significantly associated with lower 6-month mortality in patients (34). Furthermore, subgroup analyses found that the 1-year mortality risk decreased significantly with an increased Cr/CysC ratio for patients with gastrointestinal cancer, which was similar to the results of the main analysis. Similarly, another prospective cohort study of advanced non-small cell lung cancer found that patients in the sarcopenic group had a significantly lower survival time than patients in the non-sarcopenic group (17.12 vs. 23.0%, *p* = 0.004) (19). In addition, this association between Cr/CysC ratio and patient prognosis has also been noted in other diseases (35–37). The results of this study showed that RFS was significantly higher in the high serum Cr/CysC group than in the low serum Cr/CysC group based on the optimal cut-off definition (92.72 vs. 72.46%, *p* < 0.001), suggesting that the results of the Cr/CysC ratio can also be used to predict prognosis in GIST patients.

Presently, the risk of postoperative recurrence in GIST patients is usually assessed by tumour-specific factors, such as tumour diameter, risk classification, and tumour mutation type. However, our previous study found that the nutritional status of GIST patients also significantly affected prognosis (38, 39). Although considerable progress has been made in the use of GIST-specific tumour factors for postoperative recurrence risk stratification, there is still a lack of validated tools for assessing the overall nutritional status of GIST patients (40, 41). In this study, the Cr/CysC ratio was strongly associated with sarcopenia, and further Cox multifactorial analysis revealed that the Cr/CysC ratio was significantly associated with recurrence-free survival time, regardless of patient age, gender, tumour diameter, and risk classification. Overall, the Cr/CysC ratio can be considered a universally applicable, readily available, and valid method for predicting the risk of recurrence in GIST patients after surgery.

This study has several limitations. First, this was a single-centre retrospective study with a small sample size and the selection of GIST patients may have been biassed, which would have limited the generalizability of the results. Second, the calculated cutoff values for serum Cr/CysC ratio were not verified in other cohorts to further confirm its validation as a convenient screening tool for sarcopenia in GIST patients. Therefore, further prospective studies with multi-centre and large-sample size are needed. Third, Cr level could be altered in patients with acute kidney injury (AKI) and chronic kidney disease (CKD). Although a previous study found that serum Cr/CysC ratio could predict muscle wasting in CKD patients (22), the optimal cutoff value found in our study for GIST patients could still be impacted by the altered renal function if patients present with AKI and/or CKD.

## Conclusion

In conclusion, this study suggests that the serum Cr/CysC ratio may be a simple, cost-effective, and efficient screening tool for sarcopenia in GIST patients. Furthermore, a lower serum Cr/CysC ratio is associated with poorer RFS, indicating its promising prognostic value for long-term survival. However, further studies with larger sample size and different patient groups are required to validate these findings.

## Data availability statement

The raw data supporting the conclusions of this article will be made available by the authors, without undue reservation.

## Ethics statement

The studies involving human participants were reviewed and approved by the authors are accountable for all aspects of the work in ensuring that questions related to the accuracy or integrity of any part of the work are appropriately investigated and resolved. All patients were informed about the adverse effects accompanying therapies and they all signed informed consent forms. All procedures performed in studies involving human participants were following the ethical standards of the institutional and/or national research committee and with the 1964 Declaration of Helsinki and its later amendments or comparable ethical standards. The study design was approved by the Ethics Committee of The Fourth Hospital of Hebei Medical University. The patients/participants provided their written informed consent to participate in this study.

## Author contributions

QZ conception and design and administrative support. PD, PY, YT, and HG: provision of study materials or patients and collection and assembly of data. PD and CS: data analysis and interpretation. All authors contributed to the manuscript writing and final approval of manuscript.
